# Surveillance for falsified and substandard medicines in Africa and Asia by local organizations using the low-cost GPHF Minilab

**DOI:** 10.1371/journal.pone.0184165

**Published:** 2017-09-06

**Authors:** Albert Petersen, Nadja Held, Lutz Heide, on behalf of the Difäm-EPN Minilab Survey Group

**Affiliations:** 1 Difäm - German Institute for Medical Mission, Tübingen, Germany; 2 Pharmaceutical Institute, Eberhard Karls-University Tübingen, Tübingen, Germany; Mahidol-Oxford Tropical Medicine Research Unit, THAILAND

## Abstract

**Background:**

Substandard and falsified medical products present a serious threat to public health, especially in low- and middle-income countries. Their identification using pharmacopeial analysis is expensive and requires sophisticated equipment and highly trained personnel. Simple, low-cost technologies are required in addition to full pharmacopeial analysis in order to accomplish widespread routine surveillance for poor-quality medicines in low- and middle-income countries.

**Methods:**

Ten faith-based drug supply organizations in seven countries of Africa and Asia were each equipped with a Minilab of the Global Pharma Health Fund (GPHF, Frankfurt, Germany), suitable for the analysis of about 85 different essential medicines by thin-layer chromatography. Each organization was asked to collect approximately 100 medicine samples from private local medicine outlets, especially from the informal sector. The medicine samples were tested locally according to the Minilab protocols. Medicines which failed Minilab testing were subjected to confirmatory analysis in a WHO-prequalified medicine quality control laboratory in Kenya.

**Results:**

Out of 869 medicine samples, 21 were confirmed to be substandard or falsified medical products. Twelve did not contain the stated active pharmaceutical ingredient (API), six contained insufficient amounts of the API, and three showed insufficient dissolution of the API. The highest proportion of substandard and falsified medicines was found in Cameroon (7.1%), followed by the Democratic Republic of Congo (2.7%) and Nigeria (1.1%). Antimalarial medicines were most frequently found to be substandard or falsified (9.5% of all antimalarials). Thin-layer chromatography according to the Minilab protocols was found to be specific and reproducible in the identification of medicines which did not contain the stated API. Since only samples which failed Minilab testing were subjected to confirmatory testing using pharmacopeial methods, this study did not assess the sensitivity of the Minilab methodology in the detection of substandard medicines, and may underestimate the prevalence of poor-quality medicines.

**Conclusions:**

Surveillance for poor-quality medicines can be carried out by local organizations in low- and middle-income countries using a simple, low-cost technology. Such surveillance can identify an important subgroup of the circulating substandard and falsified medical products and can help to prevent them from causing harm in patients. A collaboration of the national drug regulatory authorities with faith-based organizations and other NGOs may therefore represent a promising strategy towards the Sustainable Development Goal of “ensuring access to quality medicines”.

## Introduction

The United Nations have declared access to “safe, effective, quality, and affordable essential medicines” to be one of the Sustainable Development Goals in their 2030 Agenda [1]. Yet, substandard and falsified medical products [2] represent a serious problem for public health, especially in Africa, South-East Asia and Latin America [3, 4]. The reported scale of this problem and its potential effects on public health are alarming. A meta-analysis of 21 surveys in sub-Saharan Africa concluded that 35% of antimalarial medicines failed chemical analysis, and 20% were falsified [5]. Annually 120,000 deaths of under-five children may be associated with the consumption of poor-quality antimalarials in sub-Saharan Africa alone [6]. Low- and middle-income countries (LMICs) are especially affected by this problem since they often lack resources, infrastructure and trained personnel [7] to assure the quality of locally produced and imported medicines, to carry out regular surveillance for substandard and falsified medical products, and to carry out effective law enforcement measures against criminal or negligent offenders.

The American Journal of Tropical Medicine & Hygiene has recently devoted a special issue to the “Global Pandemic of Falsified Medicines” [8]. The introductory article of this special issue correctly stated that “diagnostics are at the heart of any successful epidemic response effort” [3]. Therefore, a principal component of the fight against substandard and falsified medical products is to empower the afflicted countries and health care providers to carry out surveillance for poor-quality medicines, in order to rapidly identify such medicines and to remove them from circulation before they reach the patient and cause harm.

The quality standards which pharmaceuticals must comply with, as well as the methods to prove their compliance or non-compliance, are defined in the pharmacopeias, such as the International Pharmacopeia, the United States Pharmacopeia and the British Pharmacopeia. Most commonly, the identity and the amount of the active pharmaceutical ingredient (API) are determined using high performance liquid chromatography (HPLC). For solid oral dosage forms, the dissolution of the API which is a necessary precondition for bioavailability and therefore for effectiveness is determined in a special dissolution apparatus using quantification by HPLC or ultraviolet spectroscopy. Other pharmacopeial tests include e.g. tests for uniformity of the dosage units in terms of mass and in terms of content of API, tests for friability (i.e. mechanical stability/durability of tablets) etc. The equipment required for pharmacopeial analysis, especially for HPLC, is expensive and delicate, requiring an appropriate laboratory infrastructure including an electricity supply of constant voltage and regular maintenance by skilled personnel from the manufacturer. Performing the analyses requires highly trained professionals and expensive reagents, standards and organic solvents of high purity. Therefore, in LMICs only a few laboratories exist which can carry out such analyses. Their capacity is usually very limited and does not allow for large-scale routine surveillance of medicines in the health facilities and markets. The costs for pharmacopeial analysis are very high. A recent study reported that the price for the analysis of a single medicine sample, offered by a WHO-prequalified laboratory in South Africa, was on average 1,580 US$ [9]. The cost of the commercial pharmacopeial analysis of 155 medicine samples collected during that study [9] would have been equivalent to the cost of 295,000 courses of treatment with the respective medicines. Obviously, this is unaffordable in many developing countries.

Therefore, more affordable methods for the surveillance of medicine quality are desirable. Currently, two such technologies are commercially available and widely used. One is represented by portable Raman (or near-infrared) spectroscopy instruments, such as the hand-held TruScan RM by Thermo Fisher Scientific, Waltham, Massachusetts, USA. Raman spectroscopy requires very little time and therefore only little costs for labour. However, Raman instrumentation still requires a high capital cost compared to thin layer chromatography (see below), and depends on the availability of a library of pre-recorded spectra of authentic medicines [10]. The other technology is thin-layer chromatography (TLC), usually employed in form of the Minilab of the Global Pharma Health Fund (GPHF), a charitable organization supported by the German Merck KGaA pharmaceutical company [11]. The GPHF Minilab is a field test kit for simple thin layer chromatographic analysis of the identity and the approximate amount of the active pharmaceutical ingredients. It also includes protocols for a disintegration test for tablets and capsules and for a physical inspection of dosage forms and packaging material [12, 13]. Only very limited training is required for its use. TLC analysis using the Minilab has been employed in many medicine quality studies in Africa, Asia and South America, including studies by the Promoting the Quality of Medicines Program of the United States Pharmacopeial Convention [14, 15]. The strengths and limitations of the Minilab methodology have been discussed [9, 16–18].

The Ecumenical Pharmaceutical Network (EPN) is an international faith-based network based in Nairobi, Kenya, which comprises members in 36 countries. It seeks to strengthen the faith-based pharmaceutical sector in developing countries and to improve people’s access to quality pharmaceutical services. One of the EPN member organizations is the German Institute for Medical Mission (Deutsches Institut für Ärztliche Mission; Difäm), a German faith-based NGO promoting health care in developing countries. Among other activities, Difäm aims to strengthen 15 faith-based drug supply organizations (DSOs) in different African and Asian countries who procure drugs and medical supplies for faith-based health care institutions in their respective countries. The total annual turnover of these DSOs is about 90 million US $.

Starting from 2010 and supported by funds from the German faith-based charitable organization Bread for the World, Berlin, Germany, successively each DSO was supplied with a GPHF Minilab. By the time the present study was initiated (early 2015), ten faith-based drug supply organization from six African countries and from India had each received a GPHF Minilab and appropriate training, and used the Minilab for basic quality testing of those medicines which they procured for the faith-based health-care services in their respective countries.

In order to document the possibilities and limitations of the use of the GPHF Minilab by local drug supply organizations in Africa and Asia, the current survey was initiated. Each of the ten involved organizations in Africa and Asia was asked to collect 100 medicine samples from private medicine vendors, especially informal (= non-licensed) vendors, to analyse these samples according to the Minilab manuals, and to report the results to Difäm. If samples failed Minilab testing, a confirmatory test according to pharmacopeial procedures was carried out by the WHO-prequalified quality control laboratory of the Mission for Essential Drugs and Supplies (MEDS) in Kenya [19] which is a member of the Difäm-EPN Minilab network. The purpose of pharmacopeial testing within study was to determine to which extent failures identified in Minilab testing by local organizations in Africa and India were indeed “true failures”, i.e. to confirm the specificity of Minilab testing in such settings. Only samples which failed Minilab testing were tested in the MEDS laboratory. Pharmacopeial testing of all samples would have additionally allowed to measure the sensitivity of Minilab testing, i.e. the proportion of medicines with quality deficiencies which are indeed discovered in Minilab testing. However, pharmacopeial testing of all samples would have required approximately 390,000 US$, increasing the budget required for this study 10-fold (see cost calculation in the Results section). This exceeded the available financial resources.

In total, 869 medicine samples were analysed in this study, leading to the identification of 21 medicines which were confirmed to be falsified or substandard.

## Materials and methods

### Survey period

Medicine samples were collected between April and September 2015.

### Selection of medicines for sampling and testing

The ten involved organizations were requested to collect samples of medicines for which analytical protocols existed in the GPHF Minilab manuals [12, 13]. Currently, protocols are available for the analysis of 85 APIs [20], mostly anti-infective medicines. These 85 APIs also include eight antiretroviral compounds, but these were excluded from this study due to the high cost of the reference standards. S1 Table lists all 62 APIs which were analysed in this study. All of them were investigated in form of solid oral formulations, with the single exception of ceftriaxone injections.

### Study areas

The involved organizations were asked to collect medicines in the areas where they are located. Two organizations were based in Cameroon, one in the South-West Region and the other one in the North-West-Region. Two partners were located in the eastern part (Bukavu and Bunia) of the Democratic Republic of Congo (DRC). The other African organizations were located in Ghana (Accra), Kenya (Nairobi), Nigeria (Plateau State) and Uganda (Kampala). The two partners from India were based in the regions of Odisha and Tezpur, in the east part of the country.

### Sampling design and selection of collection sites

For the selection of the medicine collection sites, convenience sampling was used. The involved organizations were requested to collect medicine samples in their respective countries from different private drug outlets, preferably from informal (= non-licensed) drug vendors e.g. at local markets or bus stations. Since the types of formal and informal drug vendors are different in each country, no further standardization of the included collection sites was attempted. In the reports supplied by the involved organizations, the terms used to describe the collection sites were heterogeneous, e.g. “drug vendor on the open market”, “pharmacy on black market” or “private shop”. The described collection sites also included outlets with were most likely licensed, e.g. “community pharmacy”. In case of Uganda, apparently also pharmaceutical wholesalers were included.

### Sample collection

When collecting medicines from informal (= non-licensed) drug vendors, a mystery shopper approach was used routinely. However, the investigators were free to use an overt approach (i.e. identifying themselves and the purpose of the study) if this was necessary, e.g. due to requirements by the local health authorities. All medicines collected were being paid for. From each sample, 150 tablets/capsules or 50 vials of the same batch were to be collected if available. Medicines were collected in their original packages whenever possible. They were transported immediately to the local laboratory and thereupon stored in a dry, cool place until analysis. If they needed to be forwarded to another organization for retesting or for confirmatory pharmacopeial testing, transport was carried out by a commercial courier service at ambient temperature.

### Testing laboratories

Analysis according to the Minilab protocol was carried out in the laboratories of each of the ten involved faith-based drug supply organizations. For each involved organization a five-day workshop had been held, training several of its coworkers in the procedures of GPHF Minilab analysis. Testing according to pharmacopeial monographs was carried out at the WHO-prequalified medicine quality control laboratory of the Mission for Essential Drugs and Supplies (MEDS) in Nairobi, Kenya [21].

### Quality tests performed and specifications used

Physical inspection, thin-layer chromatography (TLC), colour reaction testing and disintegration testing were carried out according to the manuals of the GPHF Minilab [12, 22]. The packaging and (if available) package leaflets were inspected for spelling errors and irregularities, and for consistency of batch number and expiry dates. The dosage units were visually inspected, e.g. for undamaged, unaltered surfaces and colour uniformity.

For instant-release oral dosage forms, disintegration testing was performed using six tablets or capsules. These were kept in water at 37°C with occasional shaking or stirring. Disintegration was required to occur within 30 min. If not all of the tablets disintegrated in this time, the test was repeated (total three times).

For TLC testing [12], three tablets from each sample were analysed individually. In a typical procedure, tablets were crushed and extracted with a defined volume of the solvent indicated in the Minilab protocol for the respective API. An aliquot of the supernatant was appropriately diluted with the given solvent. 2 μl of this solution were applied to a TLC plate (Merck silica gel 60 F254, 0.2 mm thickness, 5 x 10 cm) using a micro capillary. Solutions prepared from authentic standards supplied by the Global Pharma Health Fund (www.gphf.org) as part of the GPHF Minilab were applied as comparison. The TLC plate was developed in the solvent system given in the Minilab protocol for the respective API. The active pharmaceutical ingredients were visualized as described for the respective compound in the GPHF Minilab manual, in most cases first under UV light of 254 nm and subsequently by iodine vapour.

At the beginning of this survey, samples which did not pass TLC testing were directly forwarded to the laboratory of MEDS, Kenya, for confirmatory testing according to pharmacopeial procedures. Several of these samples were found to pass analysis in the MEDS laboratory. Therefore, the procedure was modified in order to save expenses for the costly pharmacopeial analysis. Samples which did not pass TLC testing by the organization which had collected the sample were subsequently forwarded to another of the involved organizations for retesting. Only if they failed again, the samples were then forwarded to the laboratory of MEDS for pharmacopeial testing. Therefore, results of a second TLC test are only available for a part of the samples and are not included into Table 1.

**Table 1:** Overview of medicine samples collected and analysed.

		Number of:							% of samples confirmed to fail pharmaco-peial tests^#^
		sam-ples repor-ted	sam-ples exclu-ded	sam-ples inclu-ded	medi-cines (brands) inclu-ded	bat-ches inclu-ded	sam-ples failing 1st TLC test	sam-ples failing pharma-copeial tests	
1	Cameroon	111	5	106	86	97	12	9	8.5%
2	Cameroon	108	2	106	74	105	11	6	5.7%
3	DR Congo	85	0	85	83	84	8	4	4.7%
4	DR Congo	98	0	98	67	82	1	1	1.0%
5	Nigeria	98	3	95	93	95	3	1	1.1%
6	Kenya	94	0	94	78	89	0	0	0%
7	Uganda	100	69	31	31	31	0	0	0%
8	Ghana	105	16	89	59	81	0	0	0%
9	India	101	0	101	64	98	0	0	0%
10	India	64	0	64	33	64	0	0	0%
Total		964	95	869	622*	816*	35	21	2.4%

^#^ Only samples which failed TLC testing were subjected to confirmatory testing using pharmacopeial methods. It is therefore possible that the true percentage of medicines failing pharmacopeial standards is higher than indicated in the last column of this table.

* Numerical addition of the numbers of medicines and batches collected by the individual organizations would result in 668 medicines and 826 batches. The indicated total numbers of medicines and batches included in this study is slightly smaller since identical medicines (brands) and batches were sometimes collected by different organizations.

This study focused on TLC analysis. If a sample passed TLC testing but failed colour reaction testing, disintegration testing or physical inspection, the result was recorded but the sample was not forwarded to the MEDS laboratory, since funds for the costly pharmacopeial analysis were limited.

In the WHO-prequalified medicine quality control laboratory of MEDS, Kenya, the samples were analysed according to the specifications of the pharmacopeia which was indicated by the manufacturer on the product label. Typically, these tests included: identity; assay for amount of active pharmaceutical ingredients (APIs) declared on the label; dissolution of the APIs in case of solid dosage forms; and uniformity of dosage units by mass and by API content. Reference standards were obtained from the United States Pharmacopeial Convention (http://www.usp.org) and the European Directorate for the Quality of Medicines (www.edqm.eu)

The following pharmacopeial monographs were applied by MEDS:

- USP38-NF33 for amoxicillin/clavulanic acid tablets (dissolution testing according to USP36-NF31); azithromycin tablets; chloroquine phosphate tablets; mebendazole tablets; sulfadoxine/pyrimethamine tablets.
- BP 2015 for amoxicillin capsules; ampicillin capsules; captopril tablets; clomifene citrate tablets; metronidazole tablets; prednisolone tablets; quinine sulfate tablets.
- MEDS in-house methods for dihydroartemisinin/piperaquine tablets (identity, assay, dissolution).

In the laboratory of the Centers for Disease Control and Prevention (CDC), quantitative analysis of dihydroartemisinin and piperaquine in tablets was performed using a modified high performance liquid chromatographic (HPLC) procedure described by Green *et al*. [23]. Briefly, each tablet was weighed, pulverized and divided into two portions. A weighed portion was dissolved in methanol for dihydroartemisinin analysis and the other weighed portion was dissolved in 0.01 N HCl for piperaquine analysis. The solutions were sonicated and filtered prior to injection into the HPLC system. HPLC analysis was conducted using a 150 x 4.6 mm, C18, 5 micron column with the mobile phase consisting of 40% acetonitrile and 60% 0.05M perchlorate buffer (pH = 2.5) flowing through the column at 1 ml/min with detection wavelength set at 210 nm for both components.

### Definition of compliance of samples with standards

In TLC testing, the Rf (= retention factor) is the ratio of the distance travelled by the API divided by the total distance travelled by the mobile phase. Samples were considered non-compliant if the Rf value of the APIs was different by more than 10% from that of the authentic standards, and/or if the intensity of the spot was less than that of a reference containing 80% of the stated amount of the API. Before concluding non-compliance, the, TLC analysis had to be repeated twice starting from another tablet (or capsule/vial). Therefore, a negative outcome had to be observed in three independent experiments. TLC results were recorded in a standardized table. TLC plates were not photographed in some cases but not routinely.

In disintegration testing, non-compliance was concluded if, in three tests with six dosage units each, more than two out of 18 dosage units did not disintegrate in 30 min. The solid oral dosage forms included in this study did not include any slow-release or enteric-coated tablets, i.e. all were expected to disintegrate in 30 min.

In the laboratory of MEDS, definition of non-compliance followed the specifications of the respective pharmacopeial monograph. Before concluding non-compliance, the analysis was repeated by another investigator in that laboratory. Dihydroartemisinin/piperaquine tablets, which were investigated according to MEDS in-house methods, were considered non-compliant if the content of either or both APIs deviated by more than 5% from the stated amount, or if less than 70% of either or both APIs dissolved in dissolution medium in 60 min.

### Currency exchange rate

For the conversion of cost estimates from Euro to US$ the exchange rate of 1^st^ April 2015 was used (1 Euro = 1.0772 US$)

## Results

### Provision of equipment and training of personnel

The ten faith-based drug supply organization from six African countries and from India who participated in the present survey (Table 1) had been supplied with one GPHF Minilab [11] each. In a series of training workshops of five days duration each, coworkers of the collaborating DSOs were trained in the use of the Minilab. A German pharmacist acted as trainer in the first workshops, while in the subsequent trainings personnel from the previously trained organizations acted as trainers. The persons trained in the local drug supply organization for use of the Minilab were mostly pharmacists, pharmacy technicians or assistant pharmacists, and they were usually supported by an unskilled worker in the Minilab operation.

### Overview of the samples collected

**Fig 1:**
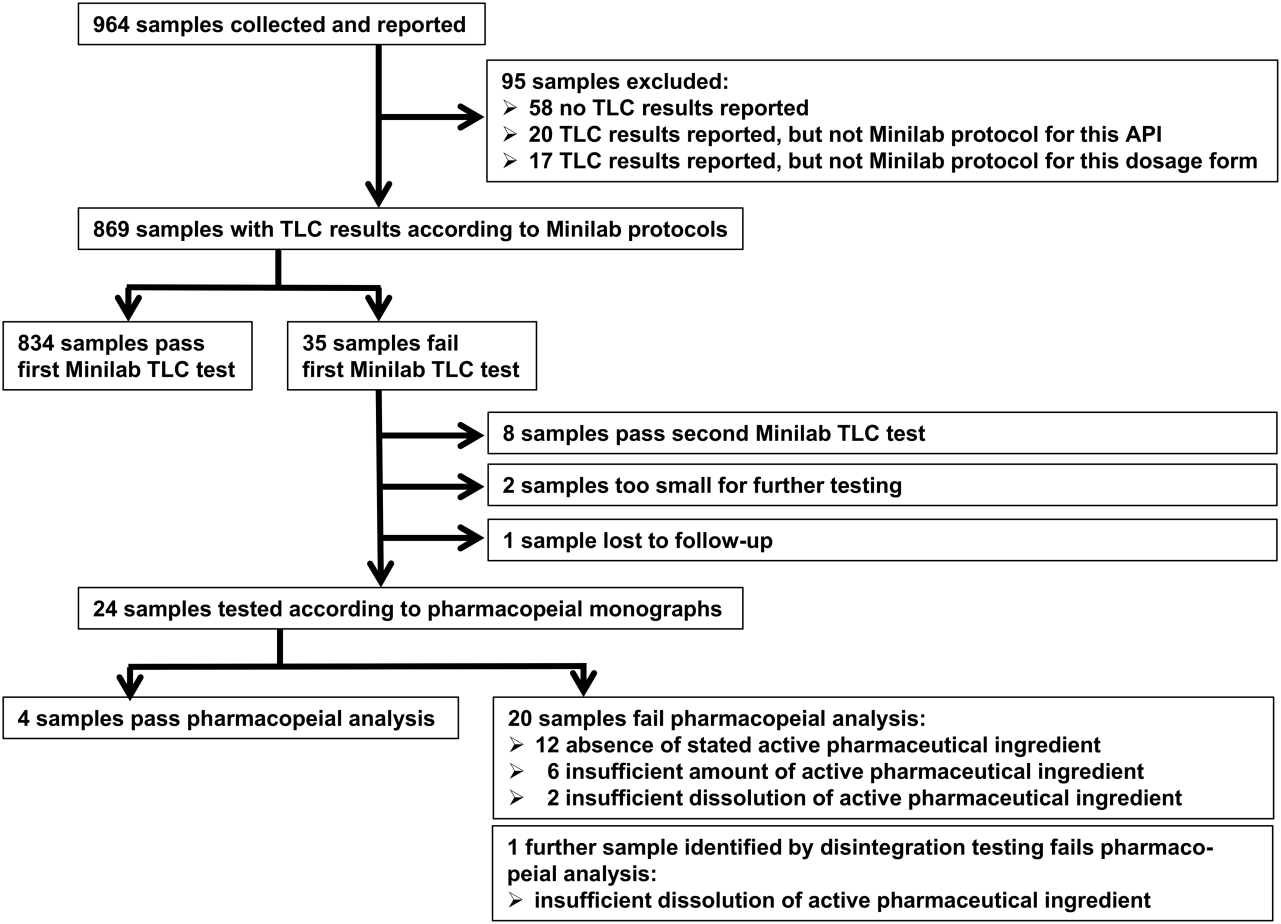
Flow chart showing sequence of analysis of medicine samples. Screening by thin layer chromatography (TLC) led to the identification of 20 samples which were confirmed to be substandard or falsified medical products. One further sample with insufficient dissolution of the active pharmaceutical ingredient was discovered from disintegration testing (see text).

As shown in Table 1, each of the ten contributing organizations collected approximately 100 samples and reported on their investigation, resulting in a total of 964 sample reports. Fig 1 shows a flow chart of the analysis of these samples during this study.

Of the 964 samples, 95 had to be excluded from data analysis. For 58 of them, no TLC results were reported. Out of these 58 cases, 54 represented medicines for which no protocol for TLC analysis was available in the manuals of the GPHF Minilab and therefore TLC analysis could not be carried out. For 4 of these 58 cases, TLC results were missing for unknown reasons.

Twenty samples were excluded from data analysis in this survey because TLC results were reported despite the fact that no Minilab protocol existed for the respective active pharmaceutical ingredient at the time of this survey (e.g. samples of amlodipine, nifedipine and ibuprofen). Seventeen further samples were excluded since TLC results were reported but no Minilab protocol existed for the respective dosage form (e.g. samples of amoxicillin suspension, ciprofloxacin i.v. infusion and metronidazole i.v. infusion). Most of these 37 TLC reports came from two of the involved organizations. Although it cannot be excluded that the investigators in these organizations developed and used their own protocols for TLC analysis, a likely explanation is that, after the sample passed visual inspection, the result “complies” was simply entered into all fields of the reporting table in these cases. This notion is supported by the observation that for 14 of the above mentioned 37 samples, the result of the disintegration test was reported as “complies”, despite the fact that a disintegration test was impossible for the respective dosage forms (e.g. oral suspensions or i.v. solutions). All 14 of these cases came from the same organization, indicating a misunderstanding of the correct procedures in the laboratory of this organization.

### WHO ATC drug classes, and generic and branded medicines

As shown in Table 1, the 869 samples included in the data analysis represented 622 branded medicines. In 45 cases, two or more samples collected in this study belonged to the same batch (as judged by the stated batch number). Therefore, the 869 samples included in the data analysis represented 816 batches of medicines.

Table 2 gives an overview of the drug classes which the sample collected in this study belonged to, using the WHO ATC classification [24]. A complete list of all 62 active pharmaceutical ingredients investigated in this study is given in S1 Table. 49% of all samples were antibacterials for systemic use, with amoxicillin tablets/capsules, ciprofloxacin tablets and cefixime tablets as the most frequent representatives (Table 2 and S1 Table). Another 12% were antiprotozoal medicines, with artemether/lumefantrine, quinine sulfate and sulfadoxine/pyrimethamine tablets most frequently encountered. Further anti-infective medicines were anthelmintics, antimycobacterials and antifungals for dermatological use. Of the latter WHO ATC category, all 21 samples were griseofulvin tablets (ATC code D01BA01). In total, anti-infective medicines represented 74.4% of the samples. Analgesics (in nearly all cases paracetamol or acetylsalicylic acid) represented 9.6% of the samples. Of the remaining 139 samples (16.0%), 38 were drugs used in diabetes (metformin and glibenclamide), 30 drugs for obstructive airways diseases (salbutamol and aminophylline) and 25 beta-blocking agents (atenolol and bisoprolol). 46 samples belonged to 4 further classes of drugs for non-communicable diseases (Table 2 and S1 Table).

**Table 2:** Overview of analysed medicine samples by drug class. No entry in a cell signifies zero.

Organization and country of collection		Drug class (WHO ATC code)													
		J01 Antibacterials for systemic use	P01 Antiprotozoals	N02, M01 Analgesics	P02 Anthelminthics	J02 Antimycobacterials	A10 Drugs used in diabetes	R03 Drugs for obstructive airway diseases	C07 Beta blocking agents	A07 Antifungals for dermatological use	C03 Diuretics	H07 Corticosteroids for systemic use	G03 Sex hormones, modulators of genital system	C09 Agents acting on the renin-angiotensin system	Total
1	Cameroon	49	14	4	7		10	5			6	4	3	4	106
2	Cameroon	52	29	6	4		1	6		4			4		106
3	DR Congo	47	4	15	10		1	1		6	1				85
4	DR Congo	45	8	17	12	2		9		5					98
5	Nigeria	47	17	6	6	2	12					5			95
6	Kenya	46	13	6	7	4	4	2	6		2	2	2		94
7	Uganda	15	3	4	1	1	2	1	2		1		1		31
8	Ghana	35	10	8	6		8	6	1	6	9				89
9	India	55	7	13	9	6			11						101
10	India	35		4	1	17			5		2				64
No. of samples per drug class		426	105	83	63	32	38	30	25	21	21	11	10	4	869
No. failing pharma-copeial analysis		4	10		2							1	3	1	21

Generic medicines sold under their international non-proprietary names (INN) constituted 310 (35.6%) of the samples. So-called branded generics, sold under a brand name given by the respective manufacturer, represented 538 (61.9%). Only 21 (2.4%) were originator brand medicines (as judged from the label claim), sold under the brand name given by the manufacturer who prepared the original new drug application for the respective API.

### Countries of origin and manufacturers

The medicines analysed in this study came from more than 290 manufacturers in 32 different countries, as judged from their labels. Table 3 gives an overview of the countries of origin. Without exception, all samples collected in India were produced in the country itself. Of the 704 samples collected in Africa, 38.1% originated from India, 18.9% from China, and 31.0% from various countries of sub-Saharan Africa. According to the label claim, 8.5% of the samples came from European countries (Table 3). Obviously, in case of falsified medicines, the country of origin stated on the label does not necessarily represent the true country of origin.

**Table 3:** Overview of analysed medicine samples by stated country of origin.

Organization and country of collection		Stated country of origin																					Total
		India	China	Ghana	Kenya	Nigeria	Uganda	DR Congo	Benin	Cameroon	South Africa	UK	Germany	France	Netherlands	Cyprus	Italy	Spain	Switzerland	Belgium	Poland	Others*	
1	Cameroon	50	33			5			3	1		5	2	3			1			1		2	106
2	Cameroon	60	14			19			1	1		1	3	4		1						2	106
3	DR Congo	25	19		20		6	9				2		1		1						2	85
4	DR Congo	47	36		1		4						1		7							2	98
5	Nigeria	34	23			31										1					1	5	95
6	Kenya	30	7		36		1				2	6	2					2				8	94
7	Uganda	14	1		2		6						2			2	1					3	31
8	Ghana	8		70								6	2						2			1	89
9	India	101																					101
10	India	64																					64
No. of samples per country of origin		433	133	70	59	55	17	9	4	2	2	20	12	8	7	5	2	2	2	1	1	25	869
No. failing pharmacopeial analysis		3	5		4		1					2	1	3		2							21

No entry in a cell signifies zero.

* Others = 11 other countries, listed in S2 Table. No out-of-specification medicines were found among these samples

### Analysis of samples and compliance with specifications

Out of the 869 samples included in the data analysis, 35 (4.0%) were reported to fail TLC testing according to the Minilab protocols, carried out by the organization who collected that sample (Fig 1). The most frequently stated reasons for failure were absence of the API (16 samples), appearance of additional spots in the TLC (seven samples) and incorrect Rf values (i.e. different chromatographic behaviour of detected compound and reference compound; two samples). For seven samples, failure of the TLC test was reported without providing further information.

Out of these 35 samples, 19 were retested in the laboratory of another of the involved organization. Eight of these samples passed the TLC retesting and were not investigated further. Of the remaining 27 samples, two could not be further investigated since too few tablets or vials were available. One sample was lost to follow-up for unknown reasons.

Therefore, 24 samples were tested in the WHO-prequalified laboratory of MEDS, Kenya, or in the laboratory of CDC in Atlanta, GA, USA. Four of these were found to comply with the pharmacopeial specifications. The remaining 20 samples were confirmed not to comply with the pharmacopeial specifications, and details of these samples are presented in Table 4.

**Table 4:** Medicine samples not complying with pharmacopeial standards.

Sam-ple no.	Country and organization of collection		Active pharmaceutical ingredient	Medicine name	Batch No.	Stated manufacturer	Stated country of origin	Confirmation of negative TLC result from:	Reason for non-compliance
1	DR Congo	3	Amoxicillin	Amoxyverse 250 mg cps	021645	Universe Pharmaceutical Ltd	Kenya	Analysis by MEDS	Absence of stated API
2	DR Congo	3	Ampicillin	Ampiverse 250 mg cps	021645	Universe Pharmaceutical Ltd	Kenya	Analysis by MEDS	Absence of stated API
3	Cameroon	1	Amoxicillin/ clavulanic acid	Augmentin 500mg/125mg tbl	448653	GlaxoSmithKline	United Kingdom	Analysis by MEDS	Absence of stated API
4	Cameroon	1	Dihydroartemisinin/ piperaquine	Duo-Cotecxin 40/320 mg tbl	031331	Zhejiang Holley Nanhu Pharmaceutical Co. Ltd	China	Analysis by CDC	Absence of stated API
5	Cameroon	1	Dihydroartemisinin/ piperaquine	Duo-Cotecxin 40/320 mg tbl	031331	Zhejiang Holley Nanhu Pharmaceutical Co. Ltd	China	Analysis by CDC	Absence of stated API
6	Cameroon	1	Dihydroartemisinin/ piperaquine	Duo-Cotecxin 40/320 mg tbl	010906	Zhejiang Holley Nanhu Pharmaceutical Co. Ltd	China	Analysis by CDC and by MEDS	Absence of stated API
7	Cameroon	2	Dihydroartemisinin/ piperaquine	Duo-Cotecxin 40/320 mg tbl	111132	Zhejiang Holley Nanhu Pharmaceutical Co. Ltd	China	Analysis by CDC	Absence of stated API
8	Cameroon	2	Sulfadoxine/ pyrimethamine	Maloxine 500/25 mg tbl	TE-3293	Gracure Pharmaceuticals Ltd	India	Analysis by MEDS	Absence of stated API
9	Cameroon	2	Sulfadoxine/ pyrimethamine	Maloxine 500/25 mg tbl	EM-304	Shreechem Laboratories	India	Analysis by MEDS	Absence of stated API
10	Nigeria	5	Quinine sulfate	Quinine sulfate 300 mg tbl	38763	Remedica Ltd	Cyprus	WHO alert No. 132/2014	Absence of stated API
11	Cameroon	2	Quinine sulfate	Quinine sulfate 300 mg tbl	38763	Remedica Ltd	Cyprus	WHO alert No. 132/2015	Absence of stated API
12	Cameroon	2	Quinine sulfate	Quinine sulfate 500 mg tbl	10H05	Novadina Pharmaceutical Ltd	UK	Analysis by MEDS and WHO alert No. 4/2016	Absence of stated API
13	DR Congo	4	Chloroquine	Chloroquine Rene	00312	Rene Industries Ltd.	Uganda	Analysis by MEDS	Assay: API only 7.1% of stated amount
14	Cameroon	1	Clomifene	Clomid 50 mg tbl	7648	Patheon France S.A,. for Aventis	France	Analysis by MEDS	Assay: API only 8.2% of stated amount
15	Cameroon	1	Clomifene	Clomid 50 mg tbl	7648	Patheon France S.A,. for Aventis	France	(same batch as sample no. 13)	(same batch as sample no. 13)
16	Cameroon	2	Clomifene	Clomid 50 mg tbl	7648	Patheon France S.A,. for Aventis	France	(same batch as sample no. 13)	(same batch as sample no. 13)
17	Cameroon	1	Captopril	Captopril 25 mg tbl	TT13152	Tuton Pharmaceuticals	India	Analysis by MEDS	Assay: API only 50.0% of stated amount
18	Cameroon	1	Prednisolone	Jpsone 5 mg tbl	130721	Jiangxi Xi'er Kangtai Pharm. Co. Ltd, for Klusyl Internat. Co. Ltd	China	Analysis by MEDS	Assay: API only 84.0% of stated amount
19	DR Congo	3	Mebendazole	Natoa 100 mg tbl	63498	Laboratory & Allied Ltd.	Kenya	Analysis by MEDS	Dissolution: 7.9% of stated amount
20	DR Congo	3	Mebendazole	Wormex 100 mg tbl	L1333	Mac' S Pharmaceuticals Ltd	Kenya	Analysis by MEDS	Dissolution: 9.3% of stated amount
21	Cameroon	1	Azithromycin	Azithromycin 500 mg tbl	131082	KIP Hamburg GmbH	Germany	Analysis by MEDS	Dissolution: 31.6% of stated amount

One additional sample was tested in the laboratory of MEDS although it had passed TLC testing, but it had failed the disintegration test according to the Minilab protocol. It was confirmed not to comply with the pharmacopeial specifications, and is listed as sample no. 21 in Table 4. The 21 samples listed in that table represent 17 different batches of medicines.

As shown in Table 4, in 12 samples the stated API was absent. Six further samples contained an insufficient amount of the API. Four of these contained even less than 10% of the stated amount of API. These included three samples of clomifene (Clomid^®^) carrying the same batch number. The Sanofi-Aventis company, contacted via the WHO, confirmed that this was a falsified medicine, showing an incorrect expiry data (P. Bourdillon Esteve, WHO, personal communication). Therefore, only one of these three Clomid^®^ samples was analysed in the laboratory of MEDS, no additional pharmacopeial analyses of the other two samples of this batch were carried out.

For samples no. 10 and 11 shown in Table 4, a WHO Medical Product Alert had already reported that the medicines with this name, manufacturer and batch number represented falsified medicines [25], therefore their analysis was not repeated in this study.

Three of the samples listed in Table 4 showed insufficient dissolution of the API, In all three cases the deviation from pharmacopeial standards was extreme.

The national drug regulatory agencies and the WHO Medical Product Alert System were informed about these findings, and warnings about several of these substandard and falsified medicines have been published on the website of the Global Pharma Health Fund [26].

### Specificity and reproducibility of TLC testing

In the present study, TLC testing using the GPHF Minilab was found to be specific and reproducible for the identification of medicines which do not contain the stated API. Sixteen samples were reported not to contain the stated API in the initial TLC analysis, and indeed fourteen of those were confirmed in pharmacopeial analysis to contain either no API or less than 10% of the stated amount of the API. For one further sample, absence of the stated API was confirmed by TLC retesting in the laboratory of another organizations, but the sample was subsequently lost to follow-up for unknown reasons (as mentioned above). Only for one of these 16 samples, retesting by TLC by another organization showed that the sample passed the retest.

Likewise, the two samples for which the collecting organization reported incorrect Rf values in TLC analysis (which implies absence of the API with the correct Rf value), were confirmed in pharmacopeial analysis not to contain the stated API in one case, and less than 10% of the stated amount of the API in the other case.

In summary, out of the 35 samples reported to fail initial TLC testing, 18 failed because the API with the correct Rf value could not be detected. Sixteen of these (Table 4, sample 1–16) were subsequently confirmed to contain either no API or less than 10% of the stated amount of the API, proving the specificity of the Minilab TLC analysis in the detection of this type of poor-quality medicines.

Seventeen samples were reported to fail initial TLC testing due to various other reasons, such as appearance of additional spots. Only four of those were subsequently confirmed not to comply with pharmacopeial specifications (Table 4, samples 17–20).

### Disintegration testing

The focus of the present study was on TLC testing. Nevertheless, disintegration testing was carried out for the solid oral dosage forms according to the Minilab protocol [12]. Out of the 869 samples included in the data analysis, 842 represented solid oral dosage forms, and for 831 of those the results of the disintegration test were reported. Forty-one of these reports stated a failure in the disintegration test. Ten of these 41 samples were retested by another of the involved organization. This showed low reproducibility: seven samples passed the retest for disintegration, while only three failed again. This may indicate that disintegration testing was not carried out under exactly equal conditions by all involved organizations, and may therefore indicate need for further training. For this reason, and due to the scarcity of funds for confirmatory pharmacopeial analysis, in this study samples which only failed disintegration testing but not TLC testing (27 samples) were not investigated further, with the single exception of the sample no. 21 (Table 4) which had passed TLC testing but failed disintegration testing and retesting.

Notably, out of the thirty-five samples which failed initial TLC testing fourteen also failed disintegration testing, showing that poor-quality medicines frequently show multiple quality deficiencies.

### Colour reaction testing

Until recently, the Minilab protocols comprised besides TLC testing also colour reaction tests for the identity of the APIs [22]. In the present study, the respective colour reaction test was carried out for 672 of the 869 investigated samples. In 21 cases, the samples were reported not to pass the colour reaction test. However, 18 of these 21 samples also did not pass the TLC test, showing a large overlap in the results of both test procedures. The Global Pharma Health Fund has discontinued the use of colour reaction tests as part of the routine Minilab protocol [27], in favour of the more specific and robust TLC testing. Our findings support the notion that inclusion of colour reaction testing provides little benefit over TLC testing alone.

### Physical inspection

**Fig 2:**
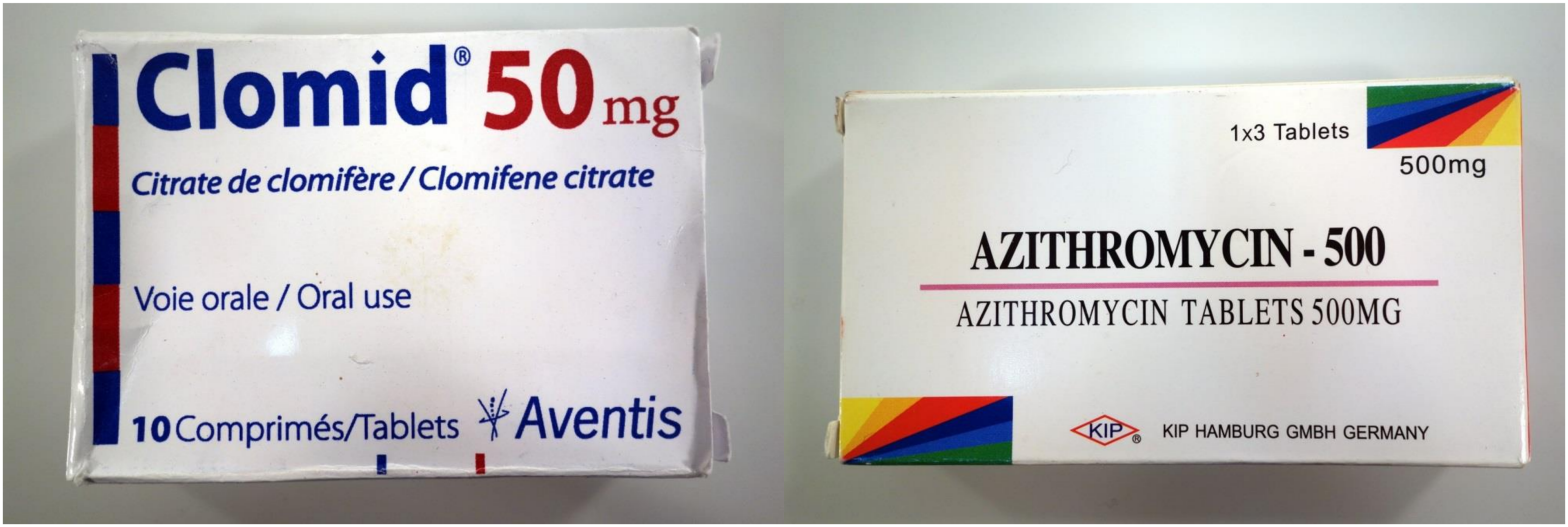
Examples of falsified medicines identified in this study. Left: Falsified Clomid tablets. Note the misspelling “Citrate de clomifère” instead of “Citrate de clomifène”. Right: Falsified Azithromycin tablets. The indicated manufacturer “KIP Hamburg GmbH Germany” does not exist. Further details of these two falsified medicines are given in Table 4 (samples no. 14 and 21).

The Minilab protocol comprises, as a first step of medicine quality investigation, a physical inspection of dosage forms and packaging materials. Visual examination of the packaging sometimes allows the immediate identification of falsified medicines from incorrect or inconsistent labelling. This may be exemplified by the two samples depicted in Fig 2.

Furthermore, visual and physical examination can detect failures in the appearance of the dosage forms (e.g. erosions, discolorations). However, as noticed in previous studies [9, 17] the outcomes of these latter assessments correlate only poorly with outcomes of pharmacopeial testing. Therefore, the most important part of physical investigation may be packaging analysis. In the present study, only ten out of 869 samples were reported not to pass visual inspection according to the procedure described in the GPHF Minilab manual [12]. Seven of these ten samples also did not pass the TLC test.

### Costing

S3 Table shows an estimate of the costs of the present survey. The total price for the purchases of the ten Minilabs, including reference standards and air freight to Africa or Asia, was 56,000 US$. The total expenses for the ten training workshops in the use of the Minilab amounted to 23,000 US$. The total external support for setting up the Minilab network was therefore approximately 79,000 US$. A certain proportion of this, e.g. 25% (19,750 US$), should be considered to be part of the costs of the present survey.

For the present survey, each of the ten involved organizations was provided with an extra budget of 1,600 US$ (for purchasing and transport costs) in order to collect and analyse approximately 100 medicine samples and to report the results to Difäm. The laboratory of the Mission for Essential Drugs and Supplies (MEDS), Nairobi, Kenya carried out confirmatory analyses for this study at a reduced price, (390–580 US$ per sample; average 450 US$ per sample), depending on the active pharmaceutical ingredient and on the tests required by the respective pharmaceutical monograph. In total, 18 pharmacopeial tests were carried out by MEDS specifically for this survey, amounting to 8,100 US$ in total. Four further samples were tested free of charge in the laboratory of the Centers for Disease Control and Prevention (CDC), Atlanta, GA, USA (courtesy of Michael D. Green).

Therefore, the total external budget support required for this survey may be estimated as approximately 43,850 US$, or 50 US$ for each medicine sample included into the data analysis. The costs for the personnel of the ten involved drug supply organizations in Africa and Asia, and of the coordinating organization in Germany, has to be considered in addition. S3 Table gives a rough estimate of the work-time required, amounting to 24.1 man-months in Africa and Asia, plus 3.5 man-months for coordination and training by Difäm, and for data analysis and report writing by Difäm and Tübingen University. The cost of the work time cannot be quantified reliably due to different local salaries and different qualification level of the involved personnel.

Even at the reduced price of 450 US $ per sample offered by MEDS for this study, the full pharmacopeial analysis of all 869 samples included into the data analysis would have costed 391,050 US $, increasing the external budget support required for this study approximately 10-fold. As mentioned in the Introduction section, a recent study reported that the price for pharmacopeial analysis of various essential medicines quoted by a WHO-prequalified laboratory in South Africa was, on average, 1,580 US $ per sample [9]. At that price, the analysis of the 869 samples of the present survey would have costed 1,373,020 US $. These figures may exemplify the need for more affordable methods for the surveillance of medicine quality in low-income countries.

## Discussion

In the present survey, 869 medicine samples from seven countries were investigated. This is a comparatively large study. Nayyar *et al*. [5], Almuzaini *et al*. [28] and Kelesidis and Falagas [29] reviewed published investigations on medicine quality in developing countries. The median numbers of samples investigated in the studies cited in these three reviews was 98, 278 and 101 samples, respectively. However, a few larger studies have been published in the last years [17, 30, 31].

In the present survey, 21 medicines (i.e. 2.4% of the 869 samples) were confirmed to be substandard or falsified medical products (Table 4). Twelve of these (i.e. 1.4% of 869 samples) did not contain the stated API, six contained an insufficient amount of the API, and three showed insufficient dissolution of the API. The GPHF Minilab is specific and sensitive in the detection of medicines which do not contain the stated API, but it is of limited sensitivity in the detection of medicines with incorrect quantity or dissolution of the API [9, 16, 17]. It is therefore possible that a number of substandard medicines escaped detection in the present study. Previous studies using full pharmacopeial analysis usually showed that the number of samples with an incorrect amount or insufficient dissolution of the API was higher than the number of samples lacking the API entirely [5, 14, 17, 28, 30].

For poor-quality medicines, WHO has discontinued the use of the misleading term “counterfeit medicines” which merely denotes an infringement of a registered trade mark, but does not consider medicine quality [32]. For a few years, WHO officially used the cumbersome term “substandard/spurious/falsely labelled/falsified/counterfeit (SSFFC) medical products”, intended as an interim solution until a consensus on a better term has been reached. Very recently, the “WHO Member State Mechanism on Substandard/Spurious/Falsely Labelled/Falsified/Counterfeit (SSFFC) Medical Products” has indeed reached such a consensus [2]. It now recommends replacing the term “SSFFC medical products” with “substandard and falsified medical products”. Notably, “substandard” and “falsified” are defined as mutually exclusive classifications [2]. Falsified medical products are “deliberately/fraudulently misrepresenting their identity, composition or source”, while substandard medical products fail to meet their quality standards or specifications for other reasons than deliberate intent, e.g. due to unintentional manufacturing mistakes, or due to degradation caused by inappropriate storage conditions. Differentiation between falsified and substandard medicines is therefore not possible on the basis of chemical analysis alone, but requires knowledge or clues of the (honest or fraudulent) intentions of the manufacturer. Such clues may be provided by packaging analysis, as exemplified by the two falsified medicines depicted in Fig 2.

If a medicine sample contains no or only a very small amount of the stated API, the likelihood that this grave mistake in production and quality control occurred without intent is very low. Therefore, the samples no. 1–16 in Table 4 may likely represent falsified medicines. Although sample no. 21 contained the correct amount of the API and failed “only” in dissolution testing, it is a falsified medicine since the stated manufacturer “KIP Hamburg GmbH Germany” (Fig 2) does not exist. To the best of our knowledge, also the stated manufacturer of samples no. 1 and 2, “Universe Pharmaceutical Ltd.”, does not exist.

Samples 17–20 failed to meet their quality standards but there is no indication whether this was due to deliberate/fraudulent intent. Unless proof of deliberate falsification is provided, these samples may therefore be classified as substandard medical products.

Within the seven countries included in this survey, the highest proportion of substandard and falsified medicines was found in Cameroon (total 15 out of 212 samples = 7.1%), followed by the Democratic Republic of Congo (2.7%) and Nigeria (1.1%). As mentioned, the true number of poor-quality medicines is likely to be even higher than detected in this study.

Comparing different therapeutic categories, the highest percentage of substandard and falsified medicines was found in antimalarial medicines (10 out of 105 samples, i.e. 9.5%). In Cameroon 8 out of 43 antimalarials (18.6%) did not contain the active pharmaceutical ingredient(s) and were therefore regarded as falsified. This figure from Cameroon is similar to the previously reported figure of 20% falsified antimalarials given by Nayyar *et al*. [5].

In the literature, the data on the prevalence of falsified medicines are highly conflicting. Some very credible studies report much lower figures than reported by Nayyar *et al*. [5]. E.g. an investigation by the World Health Organization of 935 antimalarial medicine samples from six African countries found only two samples (0.2%) in which a stated active ingredient was missing [17]. Several studies by the ACT Consortium Drug Quality Programme, mostly in West Africa, investigated a total of 10,079 antimalarial medicine samples, and only 1% of the samples were found not to contain the stated APIs [30]. These studies of the WHO [17] and of the ACT Consortium [30] sampled mostly from the formal health sector. In contrast, in the present study we tried to concentrate on the informal sector. It has been shown repeatedly that the prevalence of substandard and falsified medicines is higher in the informal sector than in the formal sector [9, 17, 28, 33, 34], and our results from Cameroon may further confirm these findings.

The prevalence of substandard and falsified medicines is known to be very different between different countries and regions. E.g. the above mentioned study by WHO [17] showed that the prevalence of poor-quality medicines was much higher in West Africa than in East Africa, and in Ethiopia no substandard or falsified antimalarial medicines were found at all. Likewise in the present survey, no substandard and falsified medicines were detected in several countries, including Kenya. For Kenya, a strong decline in the occurrence of poor-quality medicines over the period of 1997–2015 has been documented [4]. Similarly, in Malawi only a low prevalence of substandard and falsified medicines was found in public and faith-based health facilities [9]. Therefore, statements found in lay media and the internet like “30% of all medicines in developing countries are falsified” [35] are probably very misleading and should be avoided.

Table 1 shows that the numbers of samples which had to be excluded from data analysis was very high in the case from Uganda, indicating misunderstandings of the correct procedures in that organization. Furthermore, in case of Uganda many samples were apparently collected from a pharmaceutical wholesaler rather than from the informal sector. For this reason, the present survey may underestimate the prevalence of poor-quality medicines in Uganda.

Table 2 shows that the proportion of substandard and falsified medicines within the antibacterials (0.94%) was ten times lower than within the antimalarials (9.5%). Of the 139 medicines against non-communicable diseases, 5 (3.6%) were substandard or falsified. This is in agreement with earlier reports that especially antimalarial medicines are subject to falsification [5].

Regarding the stated origin of the medicines, the highest proportion of poor-quality medicines was found within the samples supposedly manufactured in Europe (13.3%), followed by samples stated to originate from China (3.8%) and from sub-Saharan Africa (1.8%) (Table 3). Within the samples stated to originate from India, only 0.7% were found to be of poor quality. As mentioned above, the country of origin stated on the label does not necessarily represent the true country of origin of the medicine. Nevertheless, the results of this study do not support the assumption that low-quality medicines found in Africa derive especially from India.

In this survey, the percentage of substandard and falsified medicines was nearly identical in generic medicines and in so-called “branded generic medicines” (1.9% and 2.0%, respectively). In contrast, within the samples claiming to be originator brand medicines, 4 of 21 (19%) were substandard or falsified, probably indicating a high propensity of originator brand medicines to become victims of criminal falsification. For patients in developing countries, the purchase of medicines labelled as originator brand medicines may therefore not present a useful strategy in order to avoid the risk of receiving substandard and falsified medicines.

The present study confirms previous reports [9, 11, 18] that simple, inexpensive TLC analysis using the GPHF Minilab is a specific and reproducible method to identify medicines which do not contain the stated API. These medicines represent an important and dangerous subgroup of substandard and falsified medical products. We recommend that within surveillance programs using TLC analysis, reporting of the analytical results should always contain a clear statement of whether or not the stated API could be detected. A medicine for which the API cannot be detected in TLC analysis using the GPHF Minilab protocol (which includes comparison to an authentic standard and three repetitions of the experiment) should not be dispensed to patients, unless a full pharmacopeial analysis proves the appropriate quality of the medicine.

When a medicine sample is reported to fail TLC analysis, photographic documentation of the TLC plate under appropriate detection (usually by UV light) provides more solid evidence than a narrative report alone. Photography of the TLC plates has been exemplified in a recent study [18]. A simple apparatus allowing easy photographic documentation of a TLC plate, using the battery-operated UV lamp supplied with the GPHF Minilab and a mobile phone camera, has recently been described [36]. This apparatus can probably be manufactured locally from plywood at minimal expense. We recommend that future surveillance programs make use of such photographic documentation of TLC analysis.

Nearly all substandard and falsified medicines identified in the present study (Table 4) showed extreme deviations from their quality standards (with the single exception of sample no. 18). This may confirm earlier reports [9, 16] that the GPHF Minilab is most powerful in the identification of medicines with extreme deviations. It has recently been suggested that the sensitivity of Minilab analysis for the detection of medicines with an incorrect amount of the API may be increased by photographic documentation combined with imaging software [36]. Proof for the practicability of this method needs to be provided by field testing.

Insufficient dissolution of the API is a frequent and serious quality problem of medicines in developing countries [5, 17, 28, 29]. In anti-infective medicines, insufficient dissolution, resulting in subtherapeutic concentrations of the medicine, may contribute to the development of resistant pathogens [37]. A WHO study [17] reported that out of 267 samples of antimalarial medicines collected in Africa, 40 samples (15%) did not pass dissolution testing according to the pharmacopeial methods. However, only 4 of these samples failed the Minilab disintegration test, showing that this test (albeit specific, simple and therefore useful) is insensitive. Simple, inexpensive pre-tests for insufficient dissolution of the API with a higher sensitivity would be useful.

Besides the GPHF Minilab, another tested and commercially available low-cost technology for medicine quality analysis is Raman (and near-infrared) spectroscopy. As mentioned in the Introduction, the identification of falsified medicines with Raman spectroscopy depends on the availability of pre-recorded reference spectra of authentic samples for each and every preparation which is to be investigated. The present survey included 622 medicines from more than 290 manufacturers in 32 countries. It would have been impossible to obtain a library of reference spectra for all these medicines before this study. Therefore, for medicine quality surveys including a large number of different medicines in developing countries, the GPHF Minilab may currently be the only commercially available ready-to-use low-cost technology.

### Limitations of this study

The central aim of the present survey was to investigate the possibilities and limitations of a surveillance of medicine quality in developing countries using the low-cost GPHF Minilab technology, carried out by the professional staff of local drug supply organizations. Inevitably, the present survey has limitations. The simple analytical methods of the GPHF Minilab are able to detect only a certain part of all possible (and relevant) quality deficiencies of medicines. Since only samples which failed Minilab testing were subjected to confirmatory testing using pharmacopeial methods, this study did not assess the sensitivity of the Minilab methodology in the detection of substandard medicines. Furthermore, the design of this multi-country study did not rigorously standardize the type of medicines to be included, or the type and location of the collection sites. Convenience sampling rather than random sampling was used in the selection of the collection sites. Therefore, in several aspects the present survey deviated from current guidelines on the conduct of surveys of the quality of medicines [38, 39], and data generated on the prevalence of substandard and falsified medicines need to be interpreted with care.

## Conclusions

In the present survey, 869 medicine samples were analysed with the GPHF Minilab, and in collaboration with a WHO-prequalified quality control laboratory, 21 samples were unequivocally confirmed to represent substandard or falsified medicines. Since only samples which failed Minilab testing were subjected to confirmatory testing using pharmacopeial methods, it is possible that additional substandard and falsified medicines were present and could not be detected with the applied methodology. Nevertheless, this study shows that surveillance for medicines which contain no or only very small amounts of the active pharmaceutical ingredient can be established at very moderate expense by local drug supply organizations using the GPHF Minilab. Such an approach can identify an important subgroup of the substandard and falsified medicines which are in circulation in developing countries. A collaboration of the national drug regulatory authorities with faith-based drug supply organizations and other NGOs, using simple low-cost analytical technologies, may represent a promising, cost-effective and as yet underutilized strategy in order to identify substandard and falsified medicines in developing countries and to prevent them from reaching the patient, in accordance with the Sustainable Development Goal to “ensure access to quality medicines”.

## Supporting information

S1 Table Overview of analysed medicine samples by drug class and active pharmaceutical ingredient. (DOCX)

S2 Table Overview of analysed medicine samples by stated country of origin: Other countries (i.e. countries not listed in Table 3). (DOCX)

S3 Table Estimate of the costs of the present survey. (DOCX)
