# Supplementary material for: Surveillance for falsified and substandard medicines in Africa and Asia by local organizations using the low-cost GPHF Minilab
Source: PLoS One. 2017 Sep 6;12(9):e0184165. doi: 10.1371/journal.pone.0184165 (PMC5587284; doi:10.1371/journal.pone.0184165)
Supplement: S1 Table — (DOCX) [file pone.0184165.s001.docx]

S1 Table: Overview of analysed medicine samples by drug class and active pharmaceutical ingredient. All medicines analysed in this study were solid oral formulations (tablets, capsules or caplets), with the single exceptions of ceftriaxone which was collected in form of injectable formulations.

| **WHO  ATC code** | **Drug class** | **Active pharmaceutical  ingredient/s** | **Number of samples** | **Percent of total [%]** |
| --- | --- | --- | --- | --- |
| C09 | Agents acting on the renin-angiotensin system | Captopril | 4 | 0.5 |
| N02 | Analgesics | Acetylsalicylic Acid | 19 | 2.2 |
| N02 | Analgesics | Paracetamol | 63 | 7.2 |
| N02/ M01 | Analgesics/Anti-inflammatory and antirheumatic products | Paracetamol/diclofenac | 1 | 0.1 |
| P02 | Anthelminthics | Albendazole | 26 | 3.0 |
| P02 | Anthelminthics | Mebendazole | 31 | 3.6 |
| P02 | Anthelminthics | Praziquantel | 6 | 0.7 |
| J01 | Antibacterials for systemic use | Amoxicillin | 54 | 6.2 |
| J01 | Antibacterials for systemic use | Amoxicillin/clavulanic acid | 13 | 1.5 |
| J01 | Antibacterials for systemic use | Ampicillin | 19 | 2.2 |
| J01 | Antibacterials for systemic use | Ampicillin/cloxacillin | 1 | 0.1 |
| J01 | Antibacterials for systemic use | Azithromycin | 26 | 3.0 |
| J01 | Antibacterials for systemic use | Cefixime | 37 | 4.3 |
| J01 | Antibacterials for systemic use | Ceftriaxone | 26 | 3.0 |
| J01 | Antibacterials for systemic use | Cefuroxime axetil | 6 | 0.7 |
| J01 | Antibacterials for systemic use | Cefalexin | 11 | 1.3 |
| J01 | Antibacterials for systemic use | Chloramphenicol | 5 | 0.6 |
| J01 | Antibacterials for systemic use | Ciprofloxacin | 49 | 5.6 |
| J01 | Antibacterials for systemic use | Clarithromycin | 3 | 0.3 |
| J01 | Antibacterials for systemic use | Cloxacillin | 26 | 3.0 |
| J01 | Antibacterials for systemic use | Doxycycline | 10 | 1.2 |
| J01 | Antibacterials for systemic use | Erythromycin | 33 | 3.8 |
| J01 | Antibacterials for systemic use | Levofloxacin | 10 | 1.2 |
| J01 | Antibacterials for systemic use | Metronidazole | 40 | 4.6 |
| J01 | Antibacterials for systemic use | Moxifloxacin | 3 | 0.3 |
| J01 | Antibacterials for systemic use | Ofloxacin | 7 | 0.8 |
| J01 | Antibacterials for systemic use | Phenoxymethylpenicillin | 8 | 0.9 |
| J01 | Antibacterials for systemic use | Sulfamethoxazole | 1 | 0.1 |
| J01 | Antibacterials for systemic use | Sulfamethoxazole/  trimethoprim | 26 | 3.0 |
| J01 | Antibacterials for systemic use | Tetracycline | 12 | 1.4 |
| D01 | Antifungals for dermatological use | Griseofulvin | 21 | 2.4 |
| J04 | Antimycobacterials | Protionamide | 1 | 0.1 |
| J04 | Antimycobacterials | Ethambutol | 12 | 1.4 |
| J05 | Antimycobacterials | Ethionamide | 1 | 0.1 |
| J04 | Antimycobacterials | Isoniazid | 2 | 0.2 |
| J04 | Antimycobacterials | Isoniazid/Rifampicin | 9 | 1.0 |
| J04 | Antimycobacterials | Pyrazinamide | 2 | 0.2 |
| J04 | Antimycobacterials | Rifampicin | 5 | 0.6 |
| P01 | Antiprotozoals | Artemether | 4 | 0.5 |
| P01 | Antiprotozoals | Artemether/Lumefantrine | 33 | 3.8 |
| P01 | Antiprotozoals | Artesunate | 1 | 0.1 |
| P01 | Antiprotozoals | Artesunate/Amodiaquine | 5 | 0.6 |
| P01 | Antiprotozoals | Atovaquone/Proguanil | 2 | 0.2 |
| P01 | Antiprotozoals | Chloroquine | 6 | 0.7 |
| P01 | Antiprotozoals | Dihydroartemisinin/Piperaquine | 6 | 0.7 |
| P01 | Antiprotozoals | Mefloquine | 2 | 0.2 |
| P01 | Antiprotozoals | Proguanil | 2 | 0.2 |
| P01 | Antiprotozoals | Quinine sulfate | 24 | 2.8 |
| P01 | Antiprotozoals | Sulfadoxine/pyrimethamine | 20 | 2.3 |
| C07 | Beta blocking agents | Atenolol | 21 | 2.4 |
| C07 | Beta blocking agents | Bisoprolol | 4 | 0.5 |
| H07 | Corticosteroids for systemic use | Prednisolone | 10 | 1.2 |
| H07 | Corticosteroids for systemic use | Prednison | 1 | 0.1 |
| C03 | Diuretics | Furosemide | 13 | 1.5 |
| C03 | Diuretics | Hydrochlorothiazide | 8 | 0.9 |
| R03 | Drugs for obstructive airway diseases | Aminophylline | 9 | 1.0 |
| R03 | Drugs for obstructive airway diseases | Salbutamol | 16 | 1.8 |
| R03 | Drugs for obstructive airway diseases | Salbutamol/theophylline | 5 | 0.6 |
| A10 | Drugs used in diabetes | Glibenclamide/metformin | 1 | 0.1 |
| A10 | Drugs used in diabetes | Glibenclamide | 13 | 1.5 |
| A10 | Drugs used in diabetes | Metformin | 24 | 2.8 |
| G03 | Sex hormones and modulators of the genital system | Clomifene | 10 | 1.2 |
| **Total** | | | 869 | 100.0 |
