# Supplementary material for: Surveillance for falsified and substandard medicines in Africa and Asia by local organizations using the low-cost GPHF Minilab
Source: PLoS One. 2017 Sep 6;12(9):e0184165. doi: 10.1371/journal.pone.0184165 (PMC5587284; doi:10.1371/journal.pone.0184165)
Supplement: S2 Table — (DOCX) [file pone.0184165.s002.docx]

S2 Table: Overview of analysed medicine samples by stated country of origin: Other countries

(i.e. countries not listed in Table 3)

| Organization  and country  of collection | | Stated country of origin | | | | | | | | | | | | |
| --- | --- | --- | --- | --- | --- | --- | --- | --- | --- | --- | --- | --- | --- | --- |
|  |  | Pakistan | Jordan | Morocco | Bangladesh | not known | Turkey | USA | Brazil | Malaysia | South Korea | Thailand | United Arab Emirates | Total others |
| 1 | Cameroon |  |  |  |  |  |  | 1 |  |  |  | 1 |  | 2 |
| 2 | Cameroon |  |  | 1 |  |  |  |  | 1 |  |  |  |  | 2 |
| 3 | DR Congo | 1 |  |  |  |  |  |  |  |  |  |  | 1 | 2 |
| 4 | DR Congo |  |  | 2 |  |  |  |  |  |  |  |  |  | 2 |
| 5 | Nigeria | 2 |  |  |  |  |  | 1 |  | 1 | 1 |  |  | 5 |
| 6 | Kenya | 3 |  |  | 2 | 1 | 2 |  |  |  |  |  |  | 8 |
| 7 | Uganda |  | 3 |  |  |  |  |  |  |  |  |  |  | 3 |
| 8 | Ghana |  |  |  |  | 1 |  |  |  |  |  |  |  | 1 |
| 9 | India |  |  |  |  |  |  |  |  |  |  |  |  | 0 |
| 10 | India |  |  |  |  |  |  |  |  |  |  |  |  | 0 |
| No. of samples  per country of origin | | 6 | 3 | 3 | 2 | 2 | 2 | 2 | 1 | 1 | 1 | 1 | 1 | 25 |
| No. failing pharmacopeial analysis | |  |  |  |  |  |  |  |  |  |  |  |  | 0 |

No entry in a cell signifies zero.
