# Supplementary material for: Surveillance for falsified and substandard medicines in Africa and Asia by local organizations using the low-cost GPHF Minilab
Source: PLoS One. 2017 Sep 6;12(9):e0184165. doi: 10.1371/journal.pone.0184165 (PMC5587284; doi:10.1371/journal.pone.0184165)
Supplement: S3 Table — (DOCX) [file pone.0184165.s003.docx]

S3 Table: Estimate of the costs of the present survey

| **A. BUDGET PROVIDED FOR THIS STUDY** | | |
| --- | --- | --- |
| **1. Costs for establishment of Minilab network (10 local drug supply organizations in Africa and Asia)** | | |
|  | - Provision of 10 GPHF Minilabs, including freight costs  (10 x 5,600 US $) | 56,000 US $ |
|  | - Organization of 10 in-country training workshops  (10 x 2,300 US $) | 23,000 US $ |
|  | *Total for establishment of network* | *79,000 US $* |
| **2. Estimated utilization of the Minilab network in the years 2010-2015** | | |
|  | - 75 % for routine basic quality testing of medicines procured by the local drug supply organization (75 % x 79,000 US $ = 59,950) |  |
|  | - 25 % for the present survey (25 % x 79,000 US $ = 19,750) |  |
|  | *Cost fraction of Minilab network required for this survey* | *19,750 US $* |
| **3. Specific costs of present survey (964 medicine samples collected; 869 of these samples included into data analysis)** | | |
|  | - Budget provided for collection, purchase of medicines and shipment of samples (10 x 1,600 US $) | 16,000 US $ |
|  | - Cost of pharmacopeial analysis at MEDS laboratory, Kenya  (18 samples x 450 US $ average cost per sample) | 8,100 US $ |
|  | *Total specific cost of this survey* | *24,100 US $* |
| **Total external budget support provided for this study:** | | |
|  | Cost fraction of Minilab network required for this study (19,750 US $) + total specific cost of this survey (24,100 US $) | **43,850 US $** |
|  | | |
| **B. ESTIMATED WORK TIME PROVIDED BY THE INVOLVED ORGANIZATIONS**  Costs of work time cannot be reliably quantified due to different local salaries and different qualification level of the involved personnel. | | |
|  | Work time for collecting and documenting of 964 samples (1 hr/sample): 964 hours |  |
|  | Work time for Minilab analysis of 964 samples (2 hr/sample): 1928 hours |  |
|  | Work time for reporting and administration (0.5 hr/sample): 482 hours |  |
| **Total worktime of local drug supply organization in Africa and Asia:**  = 3374 person hours (140 hours/month) = **24.1 man-months** | | |
|  | Work time for co-ordination by German Institute for Medical Mission = 1 man-month |  |
|  | Work time for German pharmacist as trainer in the first five in-country Minilab training workshops = 2 man-month;  of this, 25 % for the present survey = 0.5 man-months |  |
|  | Work time for data analysis and report writing = 2 man-month |  |
| **Total worktime of German personnel:**  **= 3.5 man-months** | | |
